# CD Imaging at High Spatial Resolution at Diamond B23 Beamline: Evolution and Applications

**DOI:** 10.3389/fchem.2021.616928

**Published:** 2021-04-08

**Authors:** Rohanah Hussain, Tamás Jávorfi, Giuliano Siligardi

**Affiliations:** Diamond Light Source Ltd., Harwell Science and Innovation Campus, Didcot, United Kingdom

**Keywords:** circular dichroism, CD imaging, Mueller matrix polarimetry, nano crystalline cellulose, supramolecular structure

## Abstract

Circular Dichroism imaging in the 190–650 nm spectral region pionered at Diamond Light Source B23 beamline, has been made possible by the highly collimated microbeam generated at the beamline and has been used to study the homogeneity of the supramolecular structures of thin films of chiral materials deposited on fused quartz substrates. This facility has been expanded with the installation of a Mueller Matrix Polarimeter, MMP, coupled to the beamlight, of which a preliminary data will be discussed. In the solid state, the measurement of CD related to the supramolecular structure is hampered by the presence of circular birefringence, linear dichroism, and linear birefringence that can only be evaluated using the MMP technique. The ability to characterize the chiroptical property of thin chiral films prepared under a variety of conditions and protocols such as drop cast, spin coating, spray at different temperatures and concentrations will enable the determination of the critical parameters for reproducible, uniform and homogeneous specimen preparation, which is the *sine qua non* for any commercial application. This is of particular importance for optoelectronic materials, but it can also be extended to a broad variety of materials with applications from biosensors to biological tissues.

## Introduction

Optical activity is the property of a molecule in the solid state (Arago, 1811) and in solution (Biot, 1815) to rotate the plane of polarized light that passess through it (Biot, 1815).

Circular dichroism (CD) spectroscopy is the technique most often used to characterize optically acitive chiral molecules. A molecule is chiral if its mirror image forms are nonsuperposable (Pasteur, 1849), like those of L and D amino acids. In general, any entity that is dissymmetric, namely without a plane of symmetry or a center of inversion is chiral. This applies not only to molecules but materials made of large chiral polymers, combined achiral polymers doped with small chiral molecules, and achiral polymers adopting chiral architectures.

Optically active materials show CD that is the difference between the absorption of left and right circularly polarized light passing through the sample (Cotton, 1895).

In a chromophore of a chiral molecule, an electronic transition is an helical displacement of an electron charge induced differently by the left and right circularly polarized light. The quantum-mechanical model of the one-electron mechanism describes the helical displacement of charge generated by the magnetic dipole transition moment as the rotation of charge and the electric dipole transition momement as the linear displacement of charge. Another mechanism is the exciton coupling where the helical displacement of charge is generated by the coupling of electric dipole transition moments of two twisted chromophores of the chiral molecule framework (Mason, 1978).

The different velocity at which left and right circularly polarized light travels through chiral materials is called optical rotatory dispersion (ORD) and like the counterpart CD is a manifestation of the optical activity. ORD is generated by circular birefringence that is the difference in refractive index for left and right circularly polarized light (L-cpl and R-cpl) (Fresnel, 1824).

Modern CD instruments use one photoelastic modulator (PEM) to modulate the retardation of the incident linearly polarized light, of which only the quarter wave retardation is selected (Grosjean and Legrand, 1960; Velluz et al., 1965) and measured by a photomultiplier tube (PMT) or an avalanche photo diode (APD) detector (Hussain et al., 2012). The measurement of ORD is achieved by inserting in the CD spectropolarimeter a polarizing prism, the analyser, in between the sample, and the light detector (Lowry, 1935).

**FIGURE 1:**
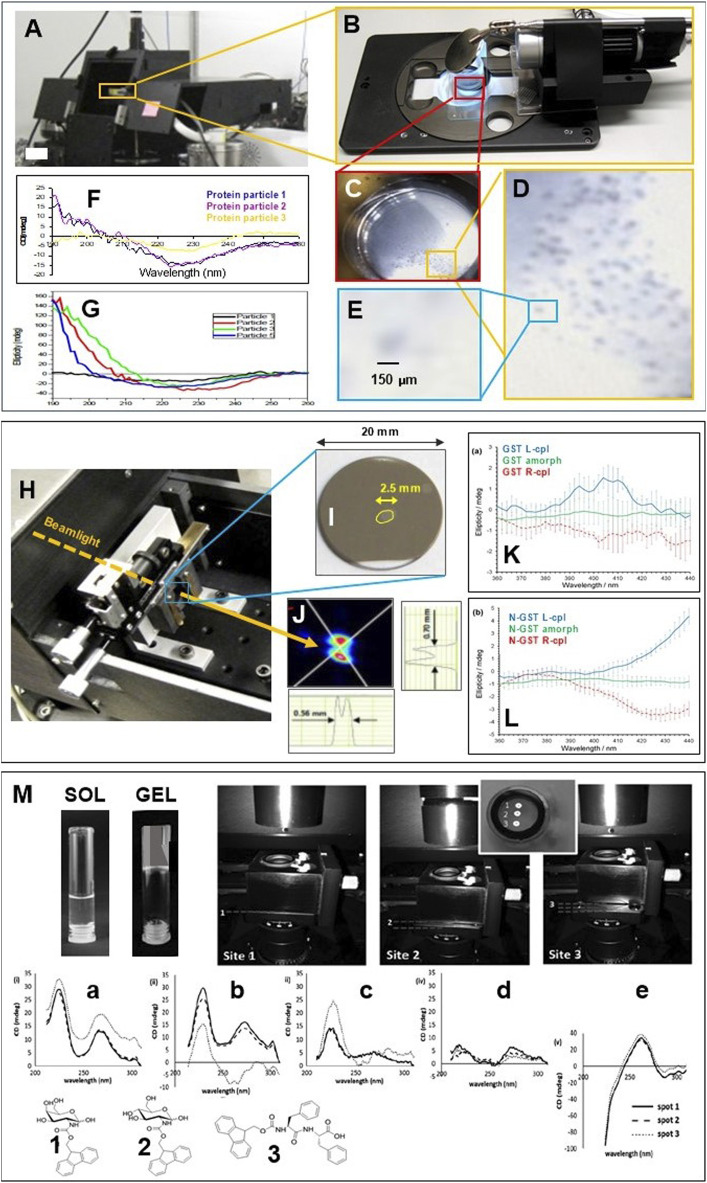
**(A)** B23 vertical sample chamber of module A. **(B)** digital camera with mirror at 45° to view the cuvette containing a suspension of protein particles in a physiological solution. **(C, E)** zommed images of cuvette cell, particles and single particle with bar size of 150 μm. **(F)** SRCD spectra of three selected particles of a β-sheet rich protein (Redrawn from 11). **(G)** a different preparation of the same proteins of **(F–H)** X-Y mechanical stage enables SRCD to be measured at different spots of the GST yellow circled area investigated using the vertical chamber of B23 module B. **(I)** Fused silica substrate window coated with a thin film of GST of which the spot of about 2.5 mm in length (circled in yellow for clarity) was sculpted with laser of left- or right-cpl. **(J)** Beamlight profile of about 600 μm in diameter at the GST specimen measured with beam profile monitor Beamage USB. **(K)** SRCD spectra of GST sculpted with L-cpl (blue), R-cpl (red) and GST outside the spot in Figure 1I, the amorphous GST (green). **(L)** SRCD spectra of N doped GST sculpted with L-cpl (red), R-cpl (red) and amorphous N-GST (green). All figures redrawn from Borisenko et al. (2015). **(M)** SRCD spectra of five gel specimens prepared from three types of samples: **(A)** GalNHFmoc (1) hydrogel in water triggered thermally (0.01 cm cell); **(B)** GalNHFmoc hydrogel (1) in PBS triggered by sonication (0.02 cm cell); **(C)** GalNHFmoc (1) hydrogel in PBS triggered thermally (0.01 cm cell); **(D)** GlcNHFmoc (2) hydrogel in water triggered thermally (0.02 cm cell); and **(E)** Fmoc‐FF (3) hydrogel in PBS triggered by sonication (0.01 cm cell). All samples were gelled in the cuvette cells at a concentration of 2.0 mg ml^−1^. For each specimen, three different positions were measured as illustrated in the insert [redrawn from Sitsanidis et al. (2018)].

The highly collimated micro beamlight generated at B23 beamline for synchroton radiation circular dichroism (SRCD) (Hussain et al., 2012) enabled the measurements of CD of solid state protein microparticles to assess the conformation homogeneity of their preparation. To our knowledge, that was the first time that CD of individual particles of 150 μm in diameter of a β-sheet rich protein was successfully measured without spectral artifacts due to light scattering and birefringence contributions (Figures 1A–G) (Gerdova, 2013). To identify and irradiate the single particles with the beamlight (Figures 1D,E), a 45° mirror to deflect the beamlight to a digital camera orthogonal to the incident light (Figure 1B) demonstrated the feasibility of this method. As the activity of proteins is strictly related to their active conformation, the production of proteins in different foldings than the active one is one of the main causes of reducing the overall protein function and activity. The ability to monitor the protein conformation under a variety of enviromental conditions is of paramount importance. It enables the optimization of the protein formulation parameters to obtain reproducible protein particles of retained native activity associated to beta strand-rich folding as illustrated in the example of the particles batch of Figure 1F compared to that in Figure 1G where significant spectral differences were observed for the same protein particles but prepared with a different method. This proof-of-principle experiment pave the way to expand and extend this approach to many other chiral materials with various applications from Life Science to Physical Sciences.

The use of an X-Y manual stage enabled the measurements of SRCD at different spots of about 600 μm in diameter of thin solid state films of pure Ge_2_Sb_2_Te_5_ (GST) and N-doped Ge_2_Sb_2_Te_5_N (N-GST) phase-change memory materials coated on fused silica substrate (Figures 1H,J; Borisenko et al., 2015). An area of about 2.5 mm in diameter of amorphous pure and N-doped GST wafer of 22 mm in diameter was pre-irradiated with L-cpl and R-cpl respectively using a powereful laser (Figure 1H) that induced a permanent optical activity of opposite chirality (Figures 1K,L) due to rapid photo-crystallization of achiral amorphous GST. The sculpted pure or N-doped achiral GST materials with L-cpl or R-cpl laser irradiation has been suggested as a potential method to control the induction of anisotropic optical properties for optoelectronic and photonic applications (Borisenko et al., 2015).

The self-assembly of three different peptide-based compounds, GalNHFmoc (1 in Figure 1M), GlcNHFmoc (2 in Figure 1M), and Fmoc‐FF (3 in Figure 1M) from sol to gel state was for the first time investigated using the B23 vertical chamber developed for high throughput CD (HTCD) (Hussain et al., 2016) to probe the homogeneity and uniformity of the supramolecular structure of the three hydrogel materials (Sitsanidis et al., 2018) of different adjacent spots of about 100 μm size. This was achieved by using a 10 × objective lens that focused the beamlight to about 100 μm in diameter on the sample without creating spectral distortions. The gelation of the peptide compounds form sol state in cylindrical cuvette cells was triggered by two methods: sonication and thermal heating and cooling cycles (Figure 1M). In an iterative manner, the method enabled the scientists to induce the gelation by varying one parameter per time in order to find out the optimum protocol for a reproducible homogeneous self-assembling. The benchtop CD instruments would give an average of a much larger area (2–3 mm in diameter) than that with B23.

The use of a 10 × objective lenses transparent down to 190 nm and a motorized X-Y stage in the B23 vertical chamber enabled the CD imaging (CDi) of thin films of chiral materials at high spatial resolution unattainable with bespoke benchtop CD instruments or any other SRCD beamline. The use of B23 CDi tower was first exploited by the Di Bari group for the characterization of the local supramolecular order in thin films of chiral functional conjugated polymers with optoelectronic properties (Zinna et al., 2017).

**FIGURE 2:**
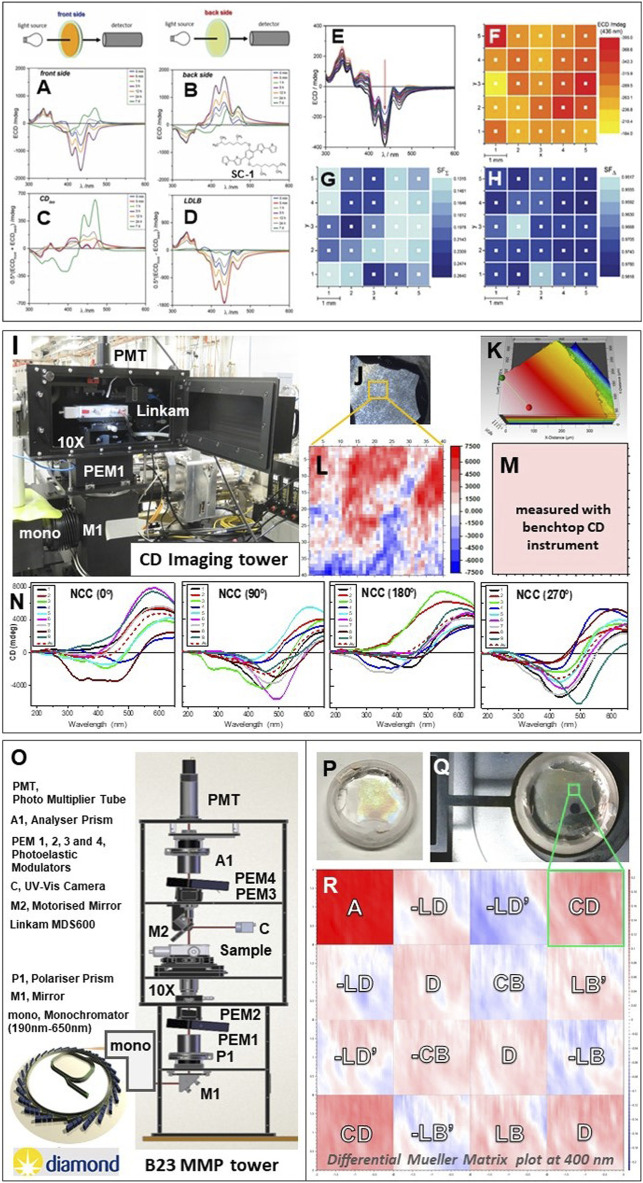
**(A)** SRCD spectra of a thin film of π-conjugated chiral molecule spin coated (SC-1) on a fused silica subtrate measured on the front side orientation as illustrated by the cartoon. **(B)** SRCD spectra of back side orientations. **(C)** Calculated SRCD spectra termed CD_iso_ from the sum of spectra [(A + B)/2]. **(D)** Calculated SRCD spectra termed LDLB from the difference of spectra [(A – B)/2]. **(E)** 25 CD spectra of front side orientation measured in a 5 × 5 grid at 1 mm intervals. **(F)** 2D map of SRCD intensities at 436 nm of the 5 × 5 grid measured in 2E. **(G)** Calculated 2D map of the CD_ISO_ intensities at 436 nm of the same 5 × 5 grid illustrated in F (the back side orientation is not shown). **(H)** Calculated 2D map of LDLB intensities at 436 nm of the 5 × 5 grid. All figures redrawn from Albano et al. (2019). **(I)** CD imaging tower (CDi) equipped with X-Y motorized Linkam temperature controlled stage MDS600 and a UV transparent 10 × objective lens to map a chiral nematic thin film of NCC **((J)** 90 μm in thickness using a Profilm 3D profilometer **(K)**. **(L)** B23 2D map of SRCD intensities of NCC front side orientation measured with a 41 × 41 grid at 50 μm interval steps for a total of 2 mm^2^ area. Note the SRCD intensity range from +7,500 (red) to −7,500 (blue) mdeg. **(M)** 2D map of CD intensity at 480 nm for the same area of 4 mm^2^ measured with Chirascan Plus CD instrument (Applied PhotoPhysics Ltd., United Kingdom). **(N)** SRCD spectra of NCC of a 3 × 3 grid at 1 mm interval steps (total area 2 mm^2^) as a function of rotation (0, 90, 180, and 270°) around the axis normal to the sample surface (front side) and parallel to the propagation direction of the incident beamlight. **(O)** Optical elements of B23 MMP tower coupled to Diamond light source from bending magnet and B23 double grating subtracting monochromator, mono; M1, 45° plane mirror; P1, linear polarizer; PEM1, PEM2, photoelastic modulators for polarization state generator; 10 ×, objective UV transparent lenses; sample holder; M2, retractible mirror for UV camera; PEM3, PEM4, photoelastic modulators for polarization state analyzer; A1, linear polarizer; PMT, photomultiplier tube detector. **(P)** Zoomed picture of the sample holder with NCC film specimen **(Q)**. **(R)** Differential Mueller Matrix plot of the 2 mm × 2 mm area with the 16 elements labeled for the corresponding physical interpretations: CD, circular dichroism; CB, circular birefringence; LD, horizontal linear dichroism; LB, horizontal linear birefringence; LD’, 45° linear dichroism; LB’, 45° linear birefringence; A, Absorbance; and D, Depolarization (D=A for materials with no depolarization). (Azzam, 1978).

A spatial resolution of 100 μm revealed an inhomogeneous supramolecular structure of the thin film of chiral 1,4-phenylene-based oligothiophene polymer (Albano et al., 2019) (Figures 2A–H). The SRCD spectra of that film measured in back side orientation showed opposite sign than the front side that was indicative of linear dichroism (LD) and linear birefringence (LB) contributions to CD and circular birefringence (CB) (Figures 2A,B). The fact that the SRCD spectral profiles of both front and back side orientations decreased in overall intensity as a function of time was consistent with a rearrangement of the supramolecular structure of the polymer. After 9 days of incubation, however, both front and back side were very similar in terms of profile shape and sign indicating a substantial reduction in LD and LB contributions. This can be better visualized with the sum of the SRCD spectra of the front and back side orientations divided by 2 that corresponds to the CD and CB contributions (Figure 2C) while the difference divided by 2 corresponds to the LD and LB elements (Albano et al., 2019) (Figure 2D).

For solutions, the solvent is the isotropic component whereas the solute being chiral is the anisotropic component that generates the CD. However, for solutions with macroscopic molecules such as large polymers and solid state films, CD, CB, LD, and LB contributions can be present and must be analyzed quantitatively with the differential Mueller matrix of data measured with MMP (Kuroda et al., 2000; Arteaga et al., 2012). In this paper the Mueller Matrix Ellipsometry is not discussed.

## Discussion

For thin films of solid state chiral materials, it is important to remember that all the polarization contributions are additive, and even without LD and LB, the remaining CB contribution will affect the spectral shape hence its accurate interpretation in CD terms.

CD measurements carried out as a function of rotation around the axis normal to the sample surface and parallel to the incident light will show spectral differences if LD and LB contributions are present.

Nanocrystalline cellulose (NNC) obtained from the hydrolysis of cotton-derived cellulose can form chiral nematic liquid crystalline phases with a left helical orientation forming iridescent chiral nematic films upon drying. The SRCD spectra of a 3 × 3 grid of 1 mm interval steps of a thin film of NCC of about 90 μm thick (Figure 2K) measured as a function of rotations at 0, 90, 180, and 270° in the B23 vertical chamber equipped with a temperature controlled motorized X-Y linear stage (Linkam MDS600) (Figure 2I) revealed unambiguosly the presence of LD and LB contributions as substantial spectral differences were observed (Figure 2N). The same area of 4 mm^2^ of the 3 × 3 grid at 1mm intervals was re-scanned as a 41 × 41 grid of 50 μm spatial resolution revealing a 2D image with spectral intensity from +7,500 to −7,500 mdeg (Figure 2L). It is important to notice that using a bespoke benchtop CD instrument (Chirascan Plus, APL, United Kingdom), the measured CD illustrated in Figure 2M was the average of the same 4 mm^2^ area that did not reveal any of the complexity of the chiroptical properties of the NCC sample of Figure 2L. Of course the 2D image measured with B23 in imaging mode (Figure 2L) is certainly containing all the four polarization contributions, CD, CB, LD, and LB, for which only the use of a Mueller Matrix Polarimeter (MMP) (Arteaga et al., 2012) would enable the extraction of all those components.

The preliminary differential Mueller matrix plot of the nanocrystalline cellulose (NCC) obtained from the hydrolysis of cotton-derived cellulose (Figures 2P,Q) measured with the new B23 MMP tower for imaging facility (Figure 2O) confirmed that indeed all polarization contributions, LD, LB, CD, and CB were present in the 2D CDi plot illustrated in Figure 2L with the use of B23 CDi tower. The CD elements of the differential Mueller Matrix were analyzed following the method developed by Arteaga of Kahr’s group (Arteaga et al., 2012) and Ossikovski group (Arwin et al., 2016). Although the two elements in the anti-diagonal representing the CD maps (Figure 2R) were both homogeneous in terms of uniform red colour associated to positive CD, the intensity magnitudes were slightly different due to depolarization (Azzam, 1978). In this case, the average between the two CD elements of the differential Mueller Matrix should be considered revealing that the same area size of 2mm x 2mm of the NCC thin film was indeed homogeneous unlike that observed with the CD imaging tower (Figure 2I) that appeared very heterogeneous (negative values in blue and positive in red (Figure 2L)). The MMP data (Figure 2R) confirmed the presence of the other CB, LD and LB components. This article is not meant to be a full characterisation of thin films of NCC that are known to have complex levels of mesoscale organisation but rather to raise the attention that, for chiral films, the use of differential Mueller Matrix is absolutely necessary in order to achieve a correct spectroscopic data interpretation. In solution, for instance, the conformation of proteins can be tuned by varying the solvent environment, pH, temperature, surfactants, ligands, and concentration and one can assess by CD spectroscopy how big these changes are and how to optimise these parameters in order to achieve reproducible protein formulations (Siligardi and Hussain, 2015). This is also occurring in the solid state, however, the chiroptical spectroscopic assessment of the supramolecular structure to be optimise because it is related to its properties, like for optoelectronic materials where a homogeneous helical arrangement of the polymer maximises the charge transfer hence the production of current (Naaman et al., 2019), would be hampered and distorted by the presence of CB, LD and LB contributions. That is why the use of the MMP to extract the CD information from the other optical properties of the solid state film is a must.

In here we have described the evolution of Diamond B23 beamline for synchrotron radiation circular dichroism from a powerful light source to push the wavelength boundaries of Xe lamps to the vacum UV spectral region to chiroptical imaging at higher spatial resolution. The new B23 MMP imaging facility (Figure 2O) is the latest tool that enables to see, and hopefully to control the chiroptical properties of materials of which properties rely on their homogeneous supramolecular structure. This will facilitate the identification of the parameters that can optimize such a homogeneity.

## Instrumentation

The SRCD measurements were conducted using two types of B23 vertical sample chambers called towers depending upon the type and size on the sample specimens (Hussain et al., 2012; Hussain et al., 2016). One is equipped with a large X-Y motorized linear stage that can accommodate large samples (maximum 12 × 8 cm^2^) operating at room temperature, and the other with an X-Y motorized and temperature controlled Linkam MDS600 stage (for a maximum of 1.5 cm × 1.5 cm sample area). The samples were held with appropriate 3D printed sample holders when necessary.

The MMP built by Hinds Instruments (United States) coupled to Diamond B23 light source and the double grating subtractive monochromator (Olis Instruments, United States) operating in the 190–750 nm region is composed of four photoelastic modulators operating simultaneously at different frequencies, two in the polarization state generator and two in the polarization state analyser (Figure 2O). The data acquisition and data processing were implemented by Hinds Instruments following the method described in Arteaga et al. (2012).

The MMP was calibrated using aqueous solution of 1S (+) 10‐Camphorsulfonic acid (5mg/ml) in a 1cm pathlength cuvette to give a positive CD intensity at 290.5nm of about 1500 mdeg. The other polarization elements of the differential Mueller Matrix were calibrated using a thin film of an optoelectronic material (F8BT:aza [P]) characterised by Mueller Matrix Ellpsometry using a Woollam RC2 (model DI) instrument and reported in Wade et al. (2020). In Wade at al. (2020), the homogeneity of the supramolecular structure of the thin film of F8BT:aza [P] was assessed at 50 micron spatial resolution by CDi using the B23 vertical chamber of Figure 2I as the size of the collimated light beam of the Woollam RC2 ellipsometer was about 3‐4 mm in diameter.

The CD spectrum from which Figure 2M was generated was measured with Chirascan Plus (Applied Photophysiscs, United Kingdom). The thickness of the thin film of NCC specimen in Figure 2K was measured with the Profilm 3D profilometer (Filmetrics, United States). The MMP was calibrated using aqueous solution of 1S (+) 10-Camphorsulfonic acid (5mg/ml) in a 1cm pathlength cuvette to give a positive CD intensity at 290.5nm of about 1500 mdeg. The other polarization elements of the differential Mueller Matrix were calibrated using a thin film of an optoelectronic material (F8BT:aza [P]) characterised by Mueller Matrix Ellpsometry using a Woollam RC2 (model DI) instrument and reported in Wade et al. (2020). In Wade at al. (2020), the homogeneity of the supramolecular structure of the thin film of F8BT:aza [P] was assessed at 50 micron spatial resolution by CDi using the B23 vertical chamber of Figure 2I as the size of the collimated light beam of the Woollam RC2 ellipsometer was about 3-4 mm in diameter.

## Data Availability

The original contributions presented in the study are included in the article/Supplementary Material, further inquiries can be directed to the corresponding author.
